# The IBLCE exam: candidate experience, motivation, study strategies used and predictors of success

**DOI:** 10.1186/s13006-018-0197-2

**Published:** 2019-01-07

**Authors:** Irena Zakarija-Grkovic, Anita Pavicic Bosnjak, Ivan Buljan, Renata Vettorazzi, Linda J. Smith

**Affiliations:** 1 Departments of Family Medicine and Clinical Skills, University of Split School of Medicine, Šoltanska 2, 21000 Split, Croatia; 2 Division of Neonatology, Department of Obstetrics and Gynaecology, Medical School University of Zagreb, University Hospital “Sveti Duh”, Zagreb, Croatia; 3 Department for Research in Biomedicine and Health, University of Split School of Medicine, Šoltanska 2, 21000 Split, Croatia; 4 University of Ljubljana, Faculty of Health Sciences, Zdravstvena pot 5, 1000 Ljubljana, Slovenia; 5 Bright Future Lactation Resource Centre, Ltd., Dayton, USA; 6 Boonshoft School of Medicine, Wright State University, Dayton, OH USA

**Keywords:** IBLCE exam, International board certified lactation consultants, Breastfeeding management, Croatia, Slovenia, Study strategies

## Abstract

**Background:**

Optimising breastfeeding rates is a public health priority. Studies have shown that all forms of extra breastfeeding support increase breastfeeding rates, including support provided by trained health professionals. International Board Certified Lactation Consultants (IBCLCs) are trained healthcare professionals in the clinical management of breastfeeding and human lactation. The IBCLC certification is a sought-after credential and can only be obtained after passing the exam administered by the International Board of Lactation Consultant Examiners (IBLCE). In Slovenia and Croatia, the IBLCE exam has been offered since 2006 and 2009, respectively. In this study, our aim was to 1) determine which candidate characteristics are associated with a passing grade on the IBLCE exam; and 2) analyse differences between candidates from Slovenia and Croatia, given Slovenians’ higher achievements in the past.

**Methods:**

In February, 2017, a 4-page, 36-question survey was sent via Survey Monkey to the available email addresses of all past IBLCE exam candidates in Croatia and Slovenia. Questions covered sociodemographic data, breastfeeding education, exam preparation, motivation and experience taking the IBLCE exam.

**Results:**

Ninety-two participants completed the online survey: 36 from Croatia and 55 from Slovenia, giving a response of 47 and 52%, respectively. No significant difference was found in pass rates between the two countries, despite Slovenians being younger and spending more time observing normal breastfeeding dyads. Variables found to be significantly more common among respondents who passed the IBLCE exam included: attending breastfeeding conferences/symposiums, using a breastfeeding atlas and studying with others. Statistical predictors of IBLCE exam success were: number of hours of bedside teaching, perceived clarity of photographs and breastfeeding conference/symposium attendance. Respondents who reported that they had attended a breastfeeding conference/symposium, had less hours of bedside teaching and perceived exam photographs as completely clear, were 7.49 (95% CI 2.26, 24.84), 0.48 (95% CI 0.28, 0.82), and 3.49 (95% CI 1.17, 10.41) times more likely to pass the exam, respectively.

**Conclusion:**

Breastfeeding conference attendance, less bedside teaching and perceived clarity of exam photographs may be predictors of IBLCE exam success. Further studies on larger samples of exam candidates are required to confirm our findings and determine other factors associated with passing the IBLCE exam.

**Electronic supplementary material:**

The online version of this article (10.1186/s13006-018-0197-2) contains supplementary material, which is available to authorized users.

## Background

Optimising breastfeeding rates is a public health priority. Numerous studies have shown that not breastfeeding has a deleterious effect on maternal and paediatric health outcomes, as well as increasing health care expenditure [1]. A recent Cochrane systematic review of 100 randomised controlled trials found that all forms of extra breastfeeding support increase breastfeeding rates, including support provided by trained health professionals [2]. International Board Certified Lactation Consultants (IBCLCs) are certified healthcare professionals in the clinical management of breastfeeding and human lactation. The availability of IBCLCs in a healthcare setting has been shown to increase breastfeeding rates, improve consumer satisfaction and trust, as well as improve an institution’s image [3]. Apart from being a clinical expert in the management of breastfeeding and human lactation, IBCLCs contribute to breastfeeding programs and policies, conduct necessary research, advocate for optimal infant and young child feeding, provide education to families and health professionals, and collaborate with all involved in the care of mothers, infants and children. To obtain the IBCLC credential, candidates must pass a rigorous exam, having satisfied the prerequisites of extensive clinical practice experience in management of lactation/breastfeeding and education in human lactation, breastfeeding and general health sciences [4].

The International Board of Lactation Consultant Examiners’ (IBLCE) examination was first administered to 250 candidates in the United States of America and simultaneously in Australia in July 1985. It is the first health-related certification to be offered internationally from its inception. As of this writing, over 50,000 candidates have taken the IBLCE exam, which has been offered in at least 21 languages and over 700 locations in more than 50 countries, with numbers rising over the last several years [5]. As of August 2016, 28,892 individuals worldwide currently hold the IBCLC credential [6]. The exam is criterion-referenced, developed by subject-matter experts, and administered in secure, proctored sites. The IBLCE is independent from all professional associations and educational institutions, thereby ensuring unbiased test development and scoring.

The first Croatian IBLCE exam was held in 2009 at the University of Split School of Medicine, Split, Croatia. The exam has been held every year since, with the exception of 2014, and has been attempted by a total of 77 candidates. Data are based on the first author’s records, kept as IBLCE country coordinator for Croatia from 2004 to 2010, and on co-author/current country coordinator’s records. All Croatian candidates who sit the exam complete the 90 h course ‘A Modern Approach to Lactation and Breastfeeding’, held annually (except in 2013) at the University of Split School of Medicine. The course was modelled on the ‘90hour Basic Lactation Course for the IBCLC Certification Exam’ held regularly in Slovenia, since 2006. Both courses are based on the Core Curriculum for Lactation Consultant Practice [7] and represent the main form of preparation for IBLCE exam candidates from Croatia and Slovenia. The first Slovenian exam was held in 2006 and has been held annually since.

The IBLCE exam pass rate among Croatian candidates has been very low in the past, with an average of 39% (range: 21–58%), whereas the pass rate among Slovenian candidates (data provided by co-author/Slovenian IBLCE Country Coordinator) has been noticeably higher (Table 1), with an average of 59% (range: 20–93%). In comparison, the average global pass rate, for the years 2009–2016, for all candidates was 85.8% (range 80–90%) and of candidates seeking initial certification 83.1% (range 79–87%) [8]. The highest pass rate in recent years was in 2010, when 87.1% of candidates for initial certification passed the examination, and lowest in 2012 and 2016 (78.9% for both years) [8]. The number of exam candidates from non-English speaking countries has been rising steadily over the years, with candidates from countries other than the United States, Canada and Australia accounting for more than 74.5% in the 2016 exam administration. Interestingly, candidates from non-English speaking countries obtained lower scores in 2016 for initial certification in comparison to their English speaking counterparts (72.5% vs. 85.5%), suggesting possible challenges for non-English speaking exam candidates [9].

**Table 1:** IBLCE exam pass rates for Slovenian, Croatian and global candidates

Year	Pass rate (%)		
	Croatian candidates	Slovenian candidates	Global candidates (overall average pass rate for initial and recertification exam)
2006		68	
2007		87	
2008		58	
2009	41	46	89
2010	58	83	90
2011	54	93	88
2012	31	43	85
2013	21	25	85
2014		60	86
2015	23	63	84
2016	43	20	80
Mean	39	59	86

Passing the IBLCE exam presents a challenge to many candidates, including those from Croatia, therefore, this study has two aims: 1) to identify factors associated with passing the IBLCE exam and 2) to compare exam candidates from Croatia and Slovenia. Our ultimate goal is to better prepare not only future candidates from Croatia and Slovenia, but all aspiring IBLCE exam candidates worldwide in achieving their goal. In addition, we are not aware of any other study that has looked at IBLCE exam candidate characteristics, exam preparation strategies and exam-taking experiences; therefore, our aim is also to address this gap in the scientific literature.

## Methods

### Design

We used a cross-sectional study design to survey IBLCE exam candidates from Croatia and Slovenia so as to obtain maximal information in minimal time from past exam candidates.

### Setting

Participants were surveyed via an online questionnaire accessible between February and March 2017. Croatia and Slovenia have a population of 4.3 and 2.1 million, respectively. Both are located in Central Europe and have similar universal, public health services, which do not currently acknowledge IBCLCs as official service providers. The Croatian Association of Lactation Consultants was founded in 2009 and there are currently 26 IBCLCs in Croatia. The Slovenian Association of Lactation Consultants was founded in 2006 and 54 IBCLCs are currently listed in Slovenia [8]. Both organisations aim to promote the IBCLC certification among relevant health care workers as the gold standard in lactation care, in line with the Blueprint for action for the protection, promotion and support of breastfeeding in Europe [10].

### Sample

Our target population was comprised of all those who had ever attempted the IBLCE exam in Croatia or Slovenia, with no exclusion criteria applied; hence, all exam candidates from 2009 to 2016 in Croatia and 2006–2016 in Slovenia were contacted. Participants were identified and contact details were obtained through collaboration with IBCLE country coordinators and the directors of the Croatian and Slovenian 90 h breastfeeding courses, both of whom are also presidents of their respective lactation consultant organisations. A total of 76 Croatian (1 candidate had emigrated) and 106 Slovenian candidates were identified.

### Measurement

We constructed a survey (see Additional file 1) consisting of a cover letter followed by 36 questions (30 closed, 6 open-ended). Two questions (Question 16 and Question 17) were from Hartwig and Dunlosky’s survey on study strategies of college students [11].We addressed the following topics: sociodemographic characteristics (including profession, age, highest qualification, place of work, duration last child breastfed, length of experience in working with breastfeeding mothers), previous breastfeeding education (formal, informal, number of hours, bed-side component), IBLCE exam preparation (reasons for applying, time spent studying, study strategies used, study materials used, usefulness of breastfeeding course) and IBCLE exam experience (clarity of instructions provided by IBLCE on application process, clarity of instructions provided by IBLCE on exam procedure, clarity of instructions given by proctors, clarity of photographs provided during exam, main obstacle to passing the exam, main facilitator to passing the exam, most difficult discipline, most difficult chronological period). Formal education referred to education obtained as part of the participant’s formal qualification, whereas informal education referred to any other breastfeeding education obtained in addition to that. At the end of the questionnaire, respondents were invited to comment on the survey and/or research topic.

### Data collection

E-mails were sent to 76 Croatian and 106 Slovenian exam candidates on February 18th and March 2^nd^2017, respectively, with a link to the survey on Survey Monkey [12]. Two reminders were sent to those who did not respond, every 7 days after the first mailing. Candidates were asked to return the completed questionnaires by 19th March, 2017. Participants were reassured that the survey was anonymous and that data would be used for research purposes only.

### Data analysis

#### Comparison between the two countries

The variables age, maximum number of hours spent studying per day and total number of days spent studying, are presented as a median (95% CI) and were tested using the Mann Whitney U test for comparison between Croatian and Slovenian participants. The survey results are presented as frequencies and percentages. We compared the answers between Croatian and Slovenian participants using Chi square. Qualitative data were not analysed in this paper.

#### Predictors of exam success analysis

We tested for differences between participants (both demographic and exam related variables) who passed the exam and those who did not, in the overall sample. Where we found significant differences, variables were used as potential predictors in logistic regression (stepwise method). For statistical analysis we used SPSS 18 (IBM Corp., released 2010, IBM SPSS Statistics for Windows, Version 18.0, Armonk, NY, USA).

### Results

#### Participant characteristics

In total, 92 participants took the online survey. One survey was incomplete (nationality was missing); hence, 91 surveys were analysed, of which 36 were from Croatia and 55 from Slovenia, giving a response of 47 and 52%, respectively. The groups were similar in most demographic variables, apart from profession and highest qualification, where a significantly greater proportion of neonatal nurses were from Slovenia, whereas more Croatian respondents had a higher education (Table 2). Slovenian participants were significantly younger compared to Croatian (Md = 41.0 years (95% CI 37.5, 45.6) vs Md = 46.5 (95% CI 41.7, 49.0); *P* = 0.034).

**Table 2:** Characteristics and experiences of Croatian and Slovenian participants (*N* = 92)^a^

Variable	Croatia	Slovenia	χ^2^	*p*
	*n* (%)			
N	36 (39.6)	55 (60.45)	3.97	0.05
Did you pass the exam?	*n* = 33	*n* = 45		
Yes	19 (57.6)	32 (71.1)	1.00	0.32
No	14 (42.4)	13 (28.9)		
Demographic characteristics				
Profession				
Community nurse	16 (41.0)	14 (24.1)	3.51	0.06
Midwife	12 (30.8)	15 (25.9)	0.38	0.54
Neonatology nurse	4 (10.3)	18 (31.0)	5.49	0.02*
Doctor	2 (5.1)	1 (1.7)	0.94	0.10
Other	5 (12.8)	10 (17.2)	0.02	0.89
Highest qualification				
University degree	9 (25.0)	14 (25.5)	7.73	0.02*
College degree	27 (75.0)	31 (56.4)		
High school degree	0 (0.0)	10 (18.2)		
Place of work				
Community health centre	15 (41.7)	19 (34.5)		
Maternity hospital	10 (27.8)	21 (38.2)	6.98	0.22
Neonatology ward	5 (11.1)	1 (1.9)		
Private practice	2 (5.6)	0 (0.0)		
Paediatric ward	1 (2.8)	2 (3.6)		
Other	4 (11.1)	11 (20.0)		
Time spent working with breastfeeding mothers (years)				
> 20	16 (44.4)	19 (34.5)	6.82	0.08
11–20	14 (38.9)	15 (27.3)		
5–10	6 (16.7)	14 (25.5)		
< 5	0 (0.0)	7 (12.7)		
Duration last child breastfed (months)			7.58	0.11
> 12	20 (57.1)	25 (47.2)		
> 6–12	6 (17.1)	17 (32.1)		
3–6	3 (8.6)	9 (17.0)		
< 3	2 (5.7)	1 (1.9)		
Did not breastfeed	4 (11.4)	1 (1.9)		
Breastfeeding education				
Was breastfeeding part of your formal education?				
Yes	15 (41.7)	33 (60.0)	2.90	0.09
No	21 (58.3)	22 (40.0)		
If yes, no. of hours				
< 10	5 (13.9)	24 (43.6)	9.55	< 0.01*
10–20	2 (5.6)	4 (7.3)		
> 20	8 (22.2)	4 (7.3)		
If yes, did it include bed-side learning?				
Yes	11 (73.3)	7 (21.9)	11.20	0.01*
No	4 (26.7)	25 (78.1)		
If yes, how many hours?				
< 5	5 (45.5)	0 (0.0)		0.08
5–10	0 (0.0)	1 (16.7)	5.58	
> 10	6 (54.5)	5 (83.3)		
Which forms of informal breastfeeding education have you attended?				
90 h IBLCE exam preparatory course	32 (88.9)	46 (83.6)	0.49	0.49
Breastfeeding conferences/symposiums	26 (72.2)	26 (47.3)	5.47	0.02*
UNICEF/WHO 20-h course for maternity staff	18 (50.0)	25 (45.5)	0.18	0.67
Breastfeeding webinars	18 (50.0)	7 (12.7)	15.00	< 0.01*
20-h course for primary health care teams	9 (25.0)	16 (29.1)	0.18	0.67
Exam preparation				
Motivation for sitting exam				
To improve my knowledge in breastfeeding	32 (88.9)	40 (72.7)	3.40	0.07
To obtain the prestigious IBCLC certificate	11 (30.6)	6 (10.9)	5.47	0.02*
So I can work as an IBCLC	10 (27.8)	14 (25.5)	0.06	0.81
To test my knowledge in breastfeeding	9 (25.0)	15 (27.3)	0.06	0.82
I was obliged to by my employer	7 (19.4)	6 (10.9)	1.28	0.26
To prove that I can do it	3 (8.3)	9 (16.4)	1.21	0.27
Study strategies used				
Reread handouts	31 (86.1)	40 (72.7)	2.25	0.13
Tested myself with practice questions	30 (83.3)	26 (47.3)	11.23	< 0.01*
Underlined or highlighted while reading	29 (80.6)	33 (60.0)	4.19	0.04*
Made outlines	14 (38.9)	28 (50.9)	1.25	0.26
Studied with others	11 (30.6)	11 (20.0)	1.31	0.25
Re-copied my notes	4 (11.1)	8 (14.5)	0.22	0.64
Crammed the night before the test	4 (11.1)	4 (7.3)	0.40	0.53
Actively participated during breastfeeding course	3 (8.3)	15 (27.3)	4.87	0.03*
Other	3 (8.3)	1 (1.8)	0.92	0.34
Materials used				
Course handouts	33 (91.7)	46 (83.6)	1.21	0.27
Translated questions from *Comprehensive Lactation Consultant Exam Review*	24 (66.7)	26 (47.3)	3.27	0.07
Breastfeeding atlas	19 (52.8)	32 (58.2)	0.26	0.61
Other	4 (11.1)	3 (5.4)	0.34	0.55
There was enough literature in my mother tongue	9 (25.0)	31 (56.4)	12.22	< 0.01*
I used study materials in English.	15 (41.7)	21 (38.2)	0.01	0.94
Study pattern used				
Study sessions spaced out over weeks/months	24 (66.7)	28 (50.9)	0.70	0.40
Most study done in the days before the exam	9 (25.0)	16 (29.1)		
Did you spend time observing mother-support groups or otherwise find time to listen to normal, healthy mothers?				
Yes	9 (25.0)	26 (47.3)	6.58	< 0.01*
No	27 (72.0)	29 (52.3)		
Exam experience				
Clarity of instructions provided by IBLCE regarding exam application process	*n* = 33	n = 45		
Completely clear	16 (48.5)	31 (68.9)	4.45	0.11
Partially clear	16 (48.5)	14 (31.1)		
Unclear	1 (3.0)	0 (0.0)		
Clarity of instructions provided by IBLCE regarding exam procedure	*n* = 32	*n* = 46		
Completely clear	19 (59.3)	40 (86.9)	8.73	< 0.01*
Partially clear	11 (34.3)	6 (13.1)		
Unclear	2 (6.4)	0 (0.0)		
Clarity of instructions provided by proctors during exam	*n* = 32	*n* = 46		
Completely clear	24 (75.0)	44 (95.6)	7.38	0.03*
Partially clear	7 (21.9)	2 (4.4)		
Unclear	1 (3.1)	0 (0.0)		
Clarity of photographs used during the exam	*n* = 33	n = 46		
Completely clear	5 (15.2)	11 (23.9)		
Partially clear	19 (57.6)	30 (65.2)	3.83	0.15
Unclear	9 (27.2)	5 (10.9)		
Was there sufficient time to solve all exam questions?	*n* = 36	*n* = 55		
Yes	27 (75.0)	43 (78.2)	2.56	0.11
No	9 (25.0)	12 (21.8)		

^a^One participant’s nationality data was missing

*Significantly lower than *p* = 0.05

When it came to breastfeeding education, fewer Croatian respondents received formal education, but of those who did, a significantly larger proportion listened to more than 20 h of breastfeeding tuition, including more hours of practical, bed-side learning (Table 2). Also, a significantly larger proportion of Croatian respondents attended breastfeeding conferences/symposiums and listened to breastfeeding webinars, as forms of informal education.

The groups were similar in their motivation for sitting the IBLCE exam (Table 2), with the majority selecting ‘to improve my knowledge in breastfeeding’. Also, there was no difference in the maximum number of hours spent studying per day (Croatian Md = 2.75 h (95% CI 2.00, 5.64) vs Slovenian Md = 3.00 h (95% CI 2.00, 3.00); *p* = 0.541). However, Croatian respondents spent significantly more days studying for the exam in total, compared to Slovenian (Md = 40.0 days (95% CI 18.8, 67.0) vs Md = 21.0 (95% CI 7.3, 30.0); *p* = 0.035).

Study strategies used varied, with more Croatian respondents testing themselves with practice questions and underlining or highlighting while reading, whereas Slovenian respondents participated more actively during the review course. Croatian respondents felt they did not have enough breastfeeding literature in their mother tongue, unlike their Slovenian counterparts. Interestingly, a significantly greater proportion of Slovenian respondents spent time observing mother- support groups or normal, healthy breastfeeding mothers as part of their exam preparation, than their Croatian colleagues. In this sample, significantly fewer Croatian respondents found the written instructions provided by IBLCE on the exam procedure or the instructions provided by the proctors on the day of the exam to be clear (Table 2), which could partly explain the difference in pass rates, even though this did not reach significance.

#### Comparison between participants who passed/failed the IBLCE exam

In the overall sample, there was no significant difference between the number of participants who passed or failed the IBLCE exam (Table 3). No difference was found either in regard to age (Md_Not passed_ = 40.5, 95% CI 35.5, 46.5 vs Md_Passed_ = 43.5, 95% CI 40.6, 48.4, *P* = 0.087), number of hours per day spent studying or number of days spent studying (Md_Not passed_ = 3, 95% CI 2, 3 vs. Md_Passed_ = 3, 95% CI 2, 4, *P* = 0.502 and Md_Not passed_ = 20.0, 95% CI 14.2, 60.0 vs. Md_Passed_ = 30.0, 95% CI 20.8, 31.22, *P* = 0.610, respectively). No significant differences were found in regard to profession, level of education, number of years spent working with breastfeeding dyads or duration of personal breastfeeding experience. However, we found significant differences in respondents’ study strategies and materials used, motivation, formal and informal education, as well as exam experience (Table 3). Respondents with less hours of formal teaching, including less bedside teaching, who had attended the 90 h exam review course, breastfeeding conferences, symposiums or webinars, whose main motivation was to improve their breastfeeding knowledge or work as an IBCLC and who prepared for the exam by using course handouts, a breastfeeding atlas, practice questions or group study were more likely to pass. Those who found the exam photographs and instructions provided by IBLCE completely clear, were also significantly more likely to pass.

**Table 3:** Characteristics of participants who passed/failed the IBLCE exam (*N* = 92)

		Passed	Failed	χ^2^	*p*
		*n* (%)	*n* (%)		
	*N*	53	39		0.18
Demographic characteristics					
Profession	Community nurse	19 (35.8)	11 (28.2)	0.60	0.44
	Midwife	14 (26.4)	13 (33.3)	0.52	0.47
	Neonatology nurse	13 (24.5)	9 (23.1)	0.03	0.87
	Doctor	3 (5.7)	0	2.28	0.13
	Other	4 (7.6)	6 (15.3)	0.73	0.40
Highest qualification	University degree	14 (27.0)	9 (23.1)	1.13	0.57
	College degree	31 (59.6)	27 (69.2)		
	High school degree	7 (13.5)	3 (7.7)		
Place of work^a^	Community health centre	21 (40.4)	13 (33.3)	7.57	0.18
	Maternity hospital	18 (34.3)	13 (33.3)		
	Neonatology ward	3 (5.8)	3 (7.7)		
	Paediatric ward	0	3 (7.7)		
	Private practice	0	2 (5.1)		
	Other	10 (19.2)	5 (12.8)		
Time spent working with breastfeeding mothers (years)	> 20	24 (46.2)	11 (28.2)	5.43	0.14
	11–20	17 (32.7)	12 (30.8)		
	5–10	9 (17.3)	11 (28.2)		
	< 5	2 (3.8)	5 (12.8)		
Duration last child breastfed (months)	> 12	27 (52.9)	18 (48.6)	0.51	0.97
	> 6–12	13 (25.5)	10 (27.0)		
	3–6	6 (11.8)	6 (16.2)		
	< 3	2 (3.9)	1 (2.7)		
	Did not breastfeed	3 (5.9)	2 (5.4)		
Breastfeeding education					
Was breastfeeding part of your formal education?	Yes	29 (55.8)	19 (48.7)	0.45	0.51
	No	23 (44.2)	20 (51.3)		
If yes, no. of hours	< 10	22 (78.6)	7 (36.8)		< 0.01*
	10–20	3 (10.7)	3 (15.8)	9.38	
	> 20	3 (10.7)	9 (47.4)		
If yes, did it include bed-side learning?	Yes	7 (17.9)	12 (30.7)	5.18	0.03*
	No	21 (53.8)	8 (20.5)		
If yes, how many hours?	< 5	2 (3.8)	3 (7.7)		0.45
	5–10	2 (3.8)	1 (2.6)	1.61	
	> 10	3 (5.7)	8 (20.5)		
Which forms of informal breastfeeding education have you attended?	90 h IBLCE exam preparatory course	53 (100.0)	26 (66.7)	20.57	< 0.01*
	Breastfeeding conferences/symposiums	42 (79.2)	11 (28.2)	23.97	< 0.01*
	UNICEF/WHO 20-h course for maternity staff	28 (52.8)	15 (38.5)	1.86	0.17
	Breastfeeding webinars	22 (41.5)	4 (10.3)	10.82	< 0.01*
	20-h course for primary health care teams	19 (35.8)	6 (15.4)	4.76	0.03*
Exam preparation					
Motivation for sitting exam	To improve my knowledge in breastfeeding	48 (90.6)	25 (64.1)	9.60	< 0.01*
	So I can work as an IBCLC	19 (35.8)	6 (15.4)	4.76	0.03*
	To test my knowledge in breastfeeding	17 (32.0)	7 (17.9)	2.24	0.13
	To obtain the prestigious IBCLC certificate	13 (24.5)	5 (12.8)	1.96	0.16
	To prove that I can do it	9 (17.0)	3 (7.7)	1.71	0.19
	I was obliged to by my employer	6 (11.3)	7 (17.9)	0.81	0.38
Study strategies used	Reread handouts	47 (88.7)	25 (64.1)	7.89	< 0.01*
	Underlined or highlighted while reading	41 (77.4)	22 (56.4)	4.56	0.03*
	Tested myself with practice questions	39 (73.6)	18 (46.2)	7.71	< 0.01*
	Made outlines	25 (47.2)	18 (46.2)	0.01	0.92
	Studied with others	19 (35.8)	4 (10.3)	7.85	< 0.01*
	Actively participated during breastfeeding course	16 (30.2)	2 (5.1)	8.99	0.01*
	Re-copied my notes	8 (15.1)	5 (12.8)	0.09	0.75
	Crammed the night before the test	7 (13.2)	1 (2.6)	3.02	0.07
Materials used^a^	Course handouts	53 (100.0)	27 (69.2)	18.75	< 0.01*
	Breastfeeding atlas	37 (69.8)	15 (38.5)	8.99	< 0.01*
	Translated questions from *Comprehensive Lactation Consultant Exam Review*	35 (66.0)	15 (38.5)	6.89	< 0.01*
There was enough literature in my mother tongue.		13 (48.1)	27 (50.9)	0.06	0.81
I used study materials in English.		16 (59.3)	24 (48.0)	0.89	0.34
Study pattern used	Study sessions spaced out over weeks/months	37 (72.5)	16 (59.3)	1.43	0.23
	Most study done in the days before the exam	14 (27.5)	11 (40.9)		
Did you spend time observing mother-support groups or otherwise find time to listen to normal, healthy mothers?	Yes	30 (56.6)	15 (55.3)	0.01	0.92
	No	23 (43.4)	12 (44.7)		
Exam experience					
Clarity of instructions provided by IBLCE regarding exam application process		*n* = 53	*n* = 26		
	Completely clear	44 (83.0)	16 (61.5)	4.41	0.11
	Partially clear	8 (15.1)	9 (34.6)		
	Unclear	1 (1.9)	1 (3.8)		
Clarity of instructions provided by IBLCE regarding exam procedure		n = 53	*n* = 27		
	Completely clear	38 (71.7)	10 (37.0)	10.26	< 0.01*
	Partially clear	14 (26.4)	17 (63.0)		
	Unclear	1 (1.9)	0		
Clarity of instructions provided by proctors during exam		*n* = 52	n = 27		
	Completely clear	47 (90.4)	22 (81.5)	2.51	0.29
	Partially clear	4 (7.7)	5 (18.5)		
	Unclear	1 (1.9)	0		
Clarity of photographs used during the exam		n = 52	n = 27		
	Completely clear	15 (28.3)	2 (7.4)		
	Partially clear	32 (60.4)	17 (63.0)	7.12	0.03*
	Unclear	6 (11.3)	8 (29.6)		
Was there sufficient time to solve all exam questions		n = 52	n = 27		
	Yes	48 (90.6)	23 (85.2)	0.52	0.47
	No	5 (9.4)	4 (14.8)		

^a^Multiple answers allowed

*Signficantly less than p = 0.05

#### Predictors of exam success

In this sample (*N* = 92), three variables were found predictive of passing the IBLCE exam: breastfeeding conference/symposium attendance (OR = 7.49, 95% CI 2.26, 24.84), number of hours of bedside teaching 0.48 (95% CI 0.28, 0.82), and perceived clarity of photographs (OR = 3.49, 95% CI 1.17, 10.41), explaining around 42% of the variance of the criteria (Table 4). Respondents who reported that they had attended a breastfeeding conference/symposium, who had found the exam photographs to be completely clear or had received fewer hours of formal bedside teaching had a higher chance of passing the exam.

**Table 4:** Results of logistic regression analysis for predicting a pass on the IBLCE exam (*N* = 78)

Criteria	Variables	Odds Ratio	95% CI for Odds ratio	R^2^*
Model 1†				
Passed the test _(0-No, 1-Yes)_	How many hours of clinical practice you had_(1-less than five to 3-more than ten)_	260.59	0.83,81,691.2	0.68
	Number of hours of bedside learning _(1-less than 5 to 3-more than 10)_	0.45	0.06,3.09	
	Forms of informal breastfeeding education have you attended: Breastfeeding conferences/symposiums_(0-No, 1-Yes)_	2.51	0.24 26.36	
	Forms of informal breastfeeding education have you attended: 20-h course for primary health care teams _(0-No, 1-Yes)_	2.55	0.97,6.71	
	Forms of informal breastfeeding education have you attended: Breastfeeding webinars_(0-No, 1-Yes)_	1.66	0.51,5.38	
	Motivation for sitting exam: To improve my knowledge in breastfeeding _(0-No, 1-Yes)_	1.42	0.58,3.44	
	Motivation for sitting exam: So I can work as an IBCLC _(0-No, 1-Yes)_	1.11	0.56,2.21	
	Study strategies used: Reread handouts _(0-No, 1-Yes)_	0.10	0.01,2.21	
	Study strategies used: Tested myself with practice questions _(0-No, 1-Yes)_	0.22	0.01,3.61	
	Study strategies used:Studied with others _(0-No, 1-Yes)_	1.12	0.54,2.34	
	Study strategies used Underlined or highlighted while reading_(0-No, 1-Yes)_	0.81	0.33,1.99	
	Materials used: Course handouts_(0-No, 1-Yes)_	0.56	0.09,3.57	
	Materials used: Translated questions from *Comprehensive Lactation Consultant Exam Review*_(0-No, 1-Yes)_	1.44	0.54,3.86	
	How clear were instructions provided by IBCLC regarding exam procedure _(1-unclear to 3-completely clear)_	1.18	0.07,19.55	
	How clear were the photographs used in the test?_(1-unclear to 3-completely clear)_	2.68	0.22,32.51	
	Constant	0.06		
Model 2‡				
Passed the test _(0-No, 1-Yes)_	How many hours of clinical practice you had_(1-less than five to 3-more than ten)_	0.48	0.28,0.82	0.42
	Non-formal breastfeeding education: *Congresses/Simposiums*_(0-No, 1-Yes)_	7.49	2.26,24.84	
	How clear were the photographs used in the test?_(1-unclear to 3-completely clear)_	3.49	1.17,10.41	
	Constant		0.08	

*Nagerlkerke R squared.

† Enter method, some variables which were significant between groups were not fitted in the model, due to the large amount of error

‡ Stepwise method, only data for significant variables were included.

## Discussion

To the best of our knowledge, this is the first study to analyse IBLCE exam preparation strategies, exam-taking experiences, candidate characteristics and predictors of exam success. In our sample, no significant difference was found in pass rates between the two countries assessed, despite Slovenians being younger and spending more time observing normal breastfeeding dyads. Several variables were found to be significantly more common among respondents who passed the IBLCE exam, regardless of nationality, including attending breastfeeding conferences/symposiums, using a breastfeeding atlas and studying with others. Based on logistic regression analysis, we found three predictors of IBLCE exam success: number of hours of bedside teaching, perceived clarity of photographs and breastfeeding conference/symposium attendance. Respondents who reported that they had attended a breastfeeding conference/symposium, who had found the exam photographs to be completely clear or had received fewer hours of formal bedside teaching had a higher chance of passing the exam.

Both the Croatian and Slovenian Lactation Consultant Associations organise regular breastfeeding conferences/symposiums, which have been held annually in Croatia since 2013 and biennially in Slovenia since 1999. These conferences provide health workers with the opportunity to hear, in their native language, the latest in breastfeeding/lactation research, update their knowledge in clinical practice, learn about novel policies and programs, as well as attend practical, skills-acquiring workshops. Therefore, it could be expected of attendees to be up-to-date in their field, confident in their skills and well informed [13], although this may depend, to a certain degree, on the content and quality of the conference. On the other hand, conference-goers tend to be intrinsically ambitious, interested in their field, keen to acquire new information and willing to learn, which may also explain why conference attendance, in our study, was a predictor of exam success. This finding, is therefore, likely to be generalizable to other settings. In addition, conferences provide a stimulating environment for “recharging one’s batteries”, as well as a unique opportunity to connect, communicate and collaborate with experts in the field. All this is conducive to learning. Those who do not have the desire or opportunity to attend conferences/symposiums, may be more likely to rely on professional textbooks, used during their formal education, when looking for breastfeeding information, although these have often been shown to be outdated, incomplete and inaccurate [14, 15].

“The quality of instruction and clinical experience that an IBCLC (or future IBCLC) receives is critical to the future of the profession.” [16]. The unexpected finding that study participants with *less* hours of bed-side (clinical) formal instruction on breastfeeding were *more* likely to pass the IBLCE exam is worrisome, as it suggests that the quality of formal breastfeeding education is less than satisfactory. This may explain why Croatian respondents, who were significantly more likely to hold a higher degree, had a lower exam pass rate than their Slovenian counterparts. A revision of the breastfeeding curriculum and literature used, as well as capacity building of teaching staff may help improve the quality of future teaching. IBCLC-supervised clinical instruction would aid in achieving this goal.

Empowering breastfeeding champions, which then serve as role models, is a key to success in protecting, promoting and supporting breastfeeding [17]. It is, therefore, no surprise that Slovenian IBLCE candidates, in our study, were predominantly neonatal nurses, given that the president of the Slovenian Lactation Consultant Association and director of the Slovenian pre-exam course is a neonatal nurse. Although no significant difference was found, noticeably more Slovenian than Croatian respondents were successful in attempting to pass the IBLCE exam, which is in line with the overall pass rates for candidates from our two countries. This could be explained by the finding that significantly more Slovenian respondents claimed to have had enough literature to study from in their native tongue and found the instructions provided by IBLCE regarding exam procedure, as well as instructions provided by proctors during the exam, to be clearer than their Croatian counterparts. Slovenian respondents were also significantly younger than Croatian, which probably influenced their ability to acquire and retain new knowledge and adapt to unfamiliar examination techniques and procedures. Another factor which may have an important role in exam preparation and success is the time spent observing normal, healthy breastfeeding dyads. The intent of one of the IBLCE exam prerequisites “clinical practice in providing care to breastfeeding families,” is to expose the candidate to normal breastfeeding, from the prenatal period through at least two years of breastfeeding, and the typical or most common challenges that mothers experience. This can be achieved by talking with mothers and babies of many ages and stages across the continuum of normal and challenging breastfeeding situations. This may partly explain the higher mean pass rate among Slovenian respondents, who spent significantly more time listening to normal, healthy mothers.

Surprisingly, no significant difference was found between respondents who passed/did not pass the IBLCE exam in regard to their profession, level of education, experience working with breastfeeding dyads or duration of personal breastfeeding experience. This is probably due to the small sample size in our study, suggesting the need for future larger studies. Our study did reveal that exam candidates who had attended an exam review course – in our sample, all those who passed the exam had completed a 90 h course - or breastfeeding conference/symposium/webinar and whose main motivation was to improve their breastfeeding knowledge were significantly more likely to pass. This information can serve as an incentive for course/conference organisers and others who provide continuous professional development activities in the field of lactation and breastfeeding.

Study strategies play an important role in student achievement. Previous studies have found that self-testing, rereading, and scheduling of study are associated with higher student achievement [11]. This has been confirmed by our study in which respondents who tested themselves with practice questions and reread handouts were significantly more likely to pass the IBLCE exam. In addition, studying with others also proved to be a study tactic more commonly found among successful exam candidates. Despite not being translated into Croatian or Slovenian, the *Breastfeeding Atlas* [18] proved to be more commonly used as a study material by respondents who passed, alongside pre-exam course handouts and translated questions from the *Comprehensive Lactation Consultant Exam Review* [19]. Given that computer-based testing is now standard procedure, factors related to clarity of instructions provided by proctors and clarity of photographs in paper-based tests are likely to become a problem of the past.

### Limitations

Despite the good response for an online survey (50%), our sample is relatively small. It appears that those more successful at the IBLCE exam participated in our study: 58% of study respondents from Croatia passed the IBLCE exam whereas an average of 39% of all past Croatian exam candidates passed, therefore, our findings may not be representative of the target population. We decided to use a level of significance less than 0.05. This may be seen as a limitation, given the high number of comparisons, which increases the risk of statistical significance when there is not a true difference between the populations being compared. However, we were unable to do a more rigorous analysis due to the small sample size. An additional issue is the limited recollection among respondents, some of whom may have sat the examination several years ago. We did not collect data on number of exam attempts or years when exams were sat, nor did we enquire about quality of exam translations, which may have affected pass rates. The timing of the pre-exam review course is another variable which may be worth investigating in future research. In Slovenia, this course is held in April, i.e. several months before the October exam; whereas in Croatia, it is held in November, i.e. almost a year prior to the IBLCE exam. Finally, we did not differentiate between on-line conferences and face-to-face conferences but assessed only conferences/symposiums that had been “attended”. Future researchers may want to assess this predictor in more detail. Similar investigations in other countries/regions would help discern the generalisability of our findings.

## Conclusion

Breastfeeding conference attendance, less bedside teaching and perceived clarity of exam photographs may be predictors of IBLCE exam success. Further studies on larger samples of exam candidates are required to confirm our findings and determine other factors associated with passing the IBLCE exam.

## Additional file

Additional file 1: IBLCE exam survey-Eng. Final. IBLCE exam survey template in English (DOCX 30 kb)

## Abbreviations

IBCLC: International board certified lactation consultant
IBLCE: International board of lactation consultant examiners
SPSS: Statistical package for the social sciences
