# Supplementary material for: The IBLCE exam: candidate experience, motivation, study strategies used and predictors of success
Source: Int Breastfeed J. 2019 Jan 7;14:2. doi: 10.1186/s13006-018-0197-2 (PMC6323655; doi:10.1186/s13006-018-0197-2)
Supplement: Supplementary file 1 — IBLCE exam survey-Eng. Final. IBLCE exam survey template in English (DOCX 30 kb) [file 13006_2018_197_MOESM1_ESM.docx]

**IBLCE EXAM PREPARATION**

**A questionnaire for IBLCE exam candidates**

Thank you for filling in this anonymous questionnaire on IBLCE exam preparation.

Please put a tick [ √ ] in the appropriate box/es.

**These questions are about yourself and your work setting.**

1. **You are a: (tick all that apply)**

1 doctor

2 midwife

3 community nurse

4 neonatal or paediatric nurse

5 other___________________________________

1. **How old are you?______**
2. **Where do you live?** 1 Croatia 2 Slovenia

1. **If you have children, how long did you breastfeed your last child?**

1 <3 mo.

2 3-6 mo.

3 >6-12 mo.

4 >12 mo.

5 I didn’t breastfeed

1. **What is your highest qualification?**

1 university degree

2 college

3 high school

1. **Where do you work?**

1 maternity hospital

2 community health service

3 neonatology ward

4 paediatric ward

5 my own practice

6 other__________________________________

1. **How long have you been working with breastfeeding mothers?**

1 < 5 years

2 5-10 years

3 11-20 years

4 > 20 years

1. **Did your undergraduate (formal) training include breastfeeding?**

1yes 2 no (*go to question 12*)

1. **If yes, how many hours of breastfeeding were included in your formal training?**

1 <10

2 10-20

3 >20

1. **Did teaching include clinical practice (bedside teaching)?** 1yes 2 no
2. **If yes, how many hours of clinical practice have you completed?**

1<5 2 5-10 3 >10

1. **Since graduating, which forms of informal breastfeeding education have you attended? (tick all that apply)**

1 UNICEF/WHO 20-h course for maternity staff

2 20-h course for primary health care teams (Breastfeeding-friendly primary care initiative)

3 80-h course ‘A Modern Approach to Lactation and Breastfeeding’

4 breastfeeding conferences/symposiums

5 webinars/breastfeeding lectures

6 other_______________________________________________________

**The following questions refer to your preparation for the IBLCE exam.**

1. **Why did you decide to apply for/sit the IBCLE exam? (*you may tick more than one response*)**

1 to test my knowledge in breastfeeding and lactation

2 to prove that I can do it

3 to obtain the prestigious IBLCE certificate

4 I was obliged to by my employer

5 so I can work as an IBCLC (in private practice, in a breastfeeding clinic,…)

6 to improve my knowledge in breastfeeding and lactation

7 other_______________________________________________________

1. **What was the maximum number of hours you spent studying in one day for the exam? __________**
2. **How many days did you spend studying for the exam in total?_____________**
3. **Which of the following best describes your pattern of study?**

1 I spaced out my study sessions over multiple weeks/months

2 I did most of my studying in the days before the test

1. **Which of the following study strategies did you use? (*tick all that apply*)**

1 test myself with questions or practice problems

2 recopied my notes

3 reread handouts, notes...

4 made outlines

5 underlined or highlighted while reading

6 studied with others

7 ˝crammed˝ lots of information the night before the test

8 asked questions or verbally participated during class

9 other________________________________________________________

1. **Which materials did you use for preparing for the exam? (*tick all that apply*)**

1 Handouts from the course ‘A Modern Approach to Lactation and Breastfeeding’

2 Breastfeeding Atlas (Wilson-Clay, Hoover)

3 Questions from ˝Comprehensive Lactation Consultant Exam Review˝ (L. Smith)

4 Other (please describe)__________________________________________

1. **Did you have sufficient materials in Croatian?** 1 yes 2 no
2. **Did you use English materials?** 1 yes 2 no (go to question 22)
3. **If yes, which?**

…………………………………………………………………………………………………………………………………….……………………………………………………………………………………………………………………………………........

1. **Did you spend time observing mother-support groups or otherwise find time to listen to normal, healthy mothers talk about breastfeeding?** 1 yes 2 no
2. **How useful was the course ‘A Modern Approach to Lactation and Breastfeeding’ in preparing for the exam?**

1 Extremely

2 Moderately

3 Slightly

4 Not at all

1. **What could be added to the course to better help prepare candidates for the IBLCE exam? (*you may tick more than one response*)**

1 More lectures

2 More practical classes

3 More case studies

4 More test questions

5 More small-group work

6 Other_______________________________________________________

**These last questions refer to your most recent experience in sitting the exam.**

1. **How clear were the instructions provided by IBLCE regarding applying for the exam?**

1 completely clear

2 partially clear

3 unclear

1. **How clear were the instructions provided by IBLCE regarding sitting the exam?**

1 completely clear

2 partly clear

3 unclear

1. **How clear were the instructions provided by the exam proctors?**

1 completely clear

2 partially clear

3 unclear

1. **Did you have enough time to answer all the exam questions?** 1 yes 2 no

1. **How clear were the photographs in the exam?**

1 completely clear

2 partially clear

3 unclear

1. **Did you pass the exam?** 1 yes (*go to question 32*) 2 no
2. **If no, what was the main obstacle to passing the exam?**__________________
3. **If yes, what was the main facilitator for passing the exam?__________________**
4. **What was your exam score?_____________________**
5. **For which discipline did you receive the lowest score?_____________________**
6. **For which chronological period did you receive the lowest score?____________**

**Would you like to make any comments on this questionnaire or on the IBLCE exam?**

**………………………………………………………………………………………………………..**

**………………………………………………………………………………………………………..**

**………………………………………………………………………………………………………**

I agree to the use of the data for research purposes.

**Many thanks for completing this anonymous questionnaire!**
